# Techniques of orthotopic renal transplantation. II. Size-matched porcine grafts in monkey recipients

**DOI:** 10.1590/ACB360503

**Published:** 2021-06-21

**Authors:** Tsuyoshi Takamura, Hiroshi Sasaki, Haruyuki Hirayama, Akihiko Kiyoshi, Makoto Inoue, Kenji Matsui, Naoto Matsumoto, Yatsumu Saito, Toshinari Fujimoto, Susumu Tajiri, Shuichiro Yamanaka, Kei Matsumoto, Takeshi Miyawaki, Takashi Yokoo, Eiji Kobayashi

**Affiliations:** 1 MD. Division of Nephrology and Hypertension – Department of Internal Medicine – The Jikei University School of Medicine – Tokyo, Japan; 2 PhD. Department of Urology – The Jikei University School of Medicine – Tokyo, Japan.; 3 MD. Department of Plastic and Reconstructive Surgery – The Jikei University School of Medicine – Tokyo, Japan.; 4 MS. Sumitomo Dainippon Pharma Co., Ltd. – Osaka, Japan.; 5 PhD. Sumitomo Dainippon Pharma Co., Ltd. – Osaka, Japan.; 6 PhD. Division of Nephrology and Hypertension – Department of Internal Medicine – The Jikei University School of Medicine – Tokyo, Japan.; 7 PhD. Department of Plastic and Reconstructive Surgery – The Jikei University School of Medicine – Tokyo, Japan.; 8 PhD. Department of Kidney Regenerative Medicine – The Jikei University School of Medicine – Tokyo, Japan.

**Keywords:** Kidney Transplantation, Surgery, Swine, Primates

## Abstract

**Purpose:**

As a classical xenotransplantation model, porcine kidneys have been transplanted into the lower abdomen of non-human primates. However, we have improved upon this model by using size-matched grafting in the orthotopic position. The beneficial aspects and surgical details of our method are reported herein.

**Methods:**

Donors were two newborn pigs (weighting 5 to 6 kg) and recipients were two cynomolgus monkeys (weighting, approximately, 7 kg). After bilateral nephrectomy, kidneys were cold-transported in Euro-Collins solution. The porcine kidney was transplanted to the site of a left nephrectomy and fixed to the peritoneum.

**Results:**

Kidneys transplanted to the lower abdomen by the conventional method were more susceptible to torsion of the renal vein (two cases). In contrast, early-stage blood flow insufficiency did not occur in orthotopic transplants of theleft kidney.

**Conclusions:**

Size-matched porcine-primate renal grafting using our method of transplanting tothe natural position of the kidneys contributes to stable post-transplant blood flow to the kidney.

## Introduction

Advancements in renal transplant therapy in the 20^th^ century involved testing new immunosuppressant therapies in primate models and developing numerous primate-to-primate transplant models^1^. Concurrently, xenotransplantation, which involves transplanting organs from donors of other species, has been researched since the beginning of organ transplant therapy. In particular, research using porcine donors has continued, as advances have been made in genetic modification technology^2^. Previous preclinical studies have used renal transplant models with porcine donors, and monkey^3^,^4^ and baboon^5^-^7^ recipients, both of which are non-human primates and thus require surgery with accurate techniques, in order to minimize the number of animals used, out of respect for animal welfare.

We have been making progress in the development of xeno-regenerative medicine, a novel branch of renal regenerative therapy in which porcine embryonic kidneys are used as a scaffold for human nephron progenitor cells in the recipient body^8^. Studies using small animals have shown that embryonic kidneys matured *in vivo* are best transplanted at the natural para-aortic location of the kidneys^9^. However, there are no models of orthotopic transplants of porcine kidneys with blood vessels in non-human primates^4^. These experimental renal transplants require anastomosis of the transplanted renal artery to be made to the abdominal aorta at a lower level than the recipient’s renal vein, as well as orthotopic anastomosis of the transplanted renal vein to the inferior vena cava^1^,^4^. Based on the experience accumulated thus far, when the recipient animal is a quadruped, the transplanted kidney hangs from the aorta or inferior vena cava, and, accordingly, the blood flow is less likely to be impaired^8^.

An earlier study of a pig-to-pig kidney transplant model described the detailed techniques of kidney grafts removed from one donor and transplanted into two recipients at the site of the left kidney^10^. In the current study, we matched the size of the kidneys between pig donors and monkey recipients, and detailed our method of transplanting the pig kidney to the natural position of the monkey’s kidneys. Further, we demonstrated improvements in terms of complications using our method compared to the conventional one.

## Methods

### Experimental animals and ethics

Donors were newborn pigs aged 20 to 28 days (weighting 5.0 to 6.12 kg). Recipients were cynomolgus monkeys aged 9 to 10 years (weighting 7.3 to 7.9 kg). Animals were treated in accordance with the *Guidelines for the Proper Conduct of Animal Experiments*. The donor procedures were approved by the IVTeC Animal Welfare Committee (Permit numbers: IVT20-26 and 20–84; trial numbers: K-20-019 and K-20-051) and performed in a facility of IVTeC Co., Ltd. (Hyogo, Japan). The transplantation experiment was conducted with the approval of the Sumitomo Dainippon Pharma Animal Ethics Committee (Animal Experiment Approval number: AN12843; trial numbers: RD-AN12843-03 and RD-AN12843-04) and performed in an experimental laboratory at Sumitomo Dainippon Pharma. Additionally, all renal xenotransplant experiments were approved by the animal ethics committee of the Jikei University School of Medicine (approval number: 2020-055). An expert surgeon (E.K), who have had more than fifty experiences of pig kidney transplantation, conducted the series of experiments^10^,^11^.

### Donor procedures

Donor pigs were housed in cages under temperature-controlled (15.0 to 28.0 ^o^C) and light-controlled conditions (12-hour light/dark cycle); pigs were provided with food (200±10 g/day) and free access to water.

**Figure 1:**
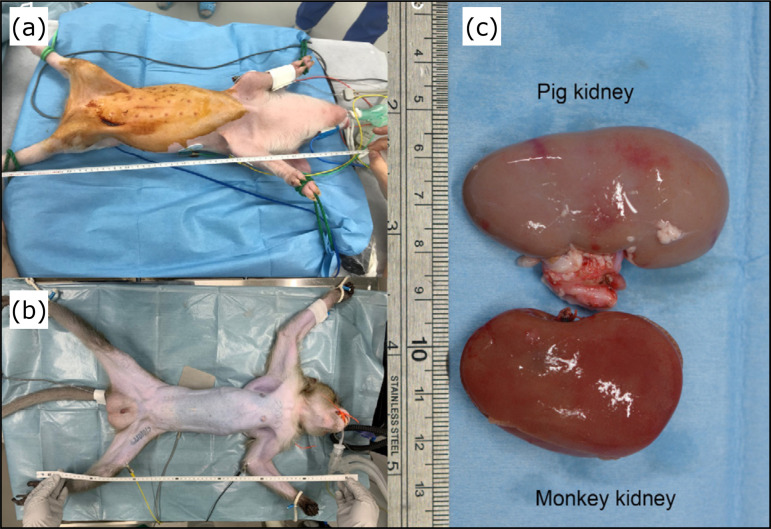
Animals used in the experiment. (a) Donor pig. (b) Recipient cynomolgus monkey. (c) Kidneys at transplantation.

The pigs were fasted for 12 h prior to surgery, with free access to water. Sedation with an intramuscular injection of a mixture of ketamine (10 mg/kg), xylazine (2.0 mg/kg), and atropine (0.25 mg/body) was followed by anesthetic induction with 5% inhalational isoflurane and 3.0 L/min oxygen. After endotracheal intubation, each pig was measured in the supine position (Fig. 1a). Anesthesia was maintained with 1 to 3% inhalational isoflurane. Although spontaneous respiration was usually retained, mechanical ventilation was used according to the depth of anesthesia. Lactated Ringer solution was dripped through an intravenous line placed in the auricular vein at a rate of 60 mL/h (adjusted according to the vital signs during the surgery).

The donor surgery was performed as previously reported^10^. Briefly, after a full-length midline abdominal intraperitoneal incision, the left kidney was mobilized by finger dissection. The perirenal tissue was then dissected to identify the left renal artery, vein, and ureter. After ligating the lumbar vein, the left renal vein could be lifted more ventrally, and the aorta could be recognized behind the left kidney. The aorta and inferior vena cava were confirmed to be exposed above the renal vessel bifurcations in the same field on the left side of the intestine. After the aorta was clamped above the renal artery bifurcation and ligated at the lower endof the dissection, a preservation solution was infused via the catheter inserted above the ligation of the aorta. The inferior vena cava was ligated at the upper and lower ends of the dissection, and then cut below and above the ligations, respectively, for a wash out. Both kidneys were procured *en bloc* while perfusing the kidneys. After adding additional perfusion at the back table, the right and left kidneys were separated. The renal arteries were then trimmed, resulting in the Carrel patch configuration. At this time, the kidneys were placed in a storage solution (Euro-Collins solution,465 mL; 50% glucose solution, 35 mL; and heparin, 1000 units) and chilled, and the removed kidneys were transported to the recipient operation facility.

### Recipient procedures

Recipient monkeys were fasted starting the evening of the day before the surgery. On the day of the surgery, an intramuscular injection of atropine (0.1 mg/kg) was administered. Anesthesia was induced with a muscular injection of ketamine (10 mg/kg) and maintained with inhaled isoflurane (0.52%). Butorphanol (0.1 mg/kg) was injected intramuscularly as a pre- and postoperative analgesic, and 47500 units/body of benzylpenicillin potassium was used as an antibiotic.

Animal size was first measured in the supine position (Fig. 1b). A midline abdominal laparotomy was performed from the xiphoid process to the pubis. Following laparotomy, the greater *omentum* and intestines were mobilized by finger dissection, and an incision was made to the peritoneum directly above the left kidney to dissect the tissues around the renal artery and left ureter, and expose the nearby abdominal artery and lower vena cava. The left renal artery, left renal vein, and left ureter were ligated to remove the left kidney. The removed left kidney was weighed, and its size was measured against the donor pig kidney (Fig. 1c).

**Figure 2:**
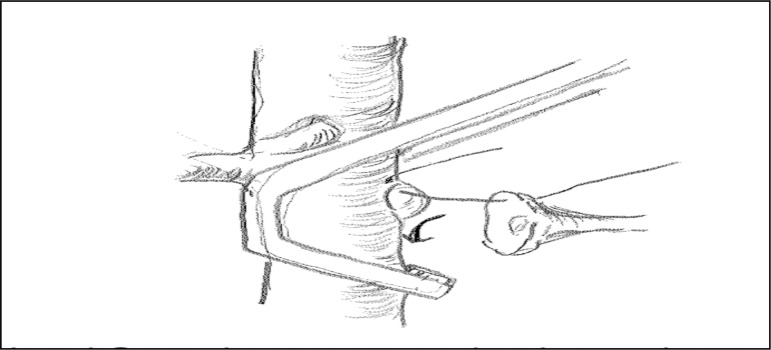
Blood flow clamping method in orthotopic transplantation. Clamp selectively below the left renal artery without clamping the mesenteric arteries or right renal artery.

**Figure 3:**
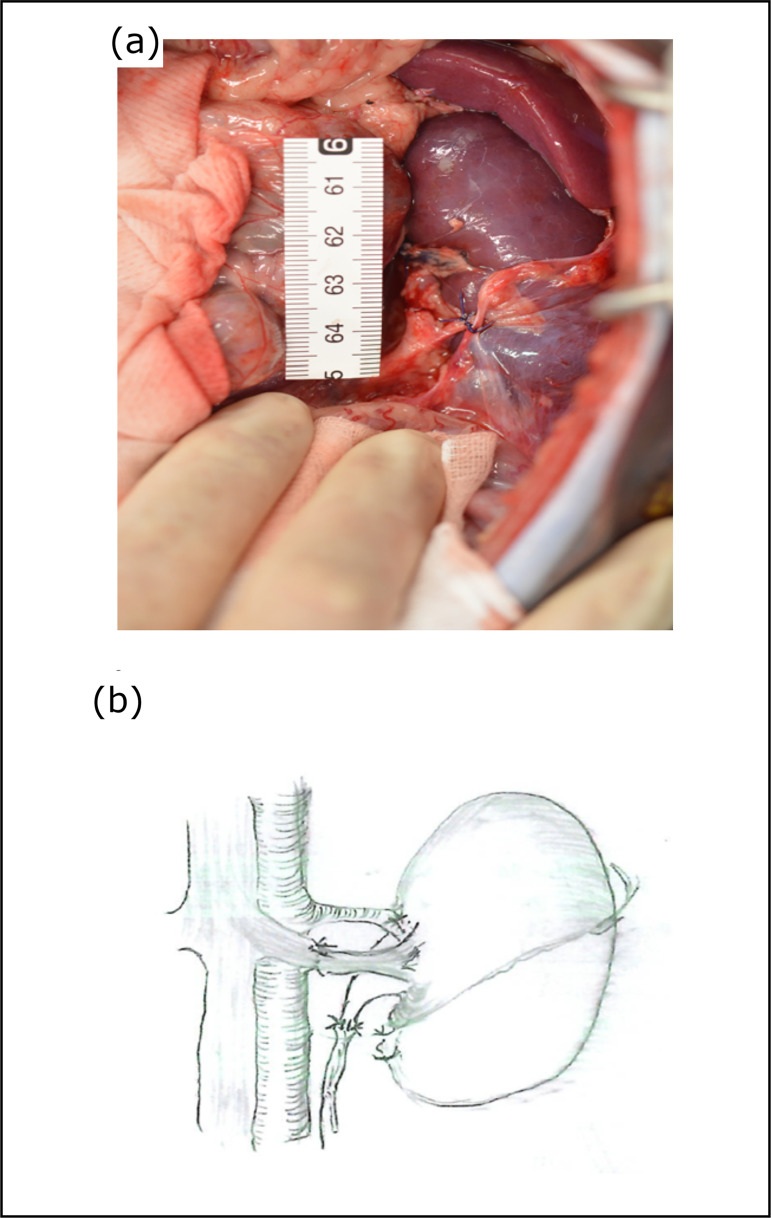
(a) Photograph during anastomosis of the pig kidney in orthotopic transplantation. (b) Diagram of suturing. Monkey and pig renal arteries and veins are sutured.

At this time, heparin (0.5 to 1.0 cc; 500 to 1000 units) was administered intravenously to the recipient. For recipients undergoing transplantation by the conventional method, the abdominal artery approximately 5 cm inferior to the left renal artery and the tissues surrounding the descending vena cava were dissected so that a Satinsky clamp could be used. The kidney was transplanted at the same site as that in a previous report^4^. For recipients undergoing the orthotopic transplantation method developed in this study, Satinsky forceps were used to clamp the aorta around the base of the renal artery so as to not clamp the mesenteric arteries or the right renal artery (Fig. 2). Sharp scissors were used to create an anastomotic opening approximately 8 mm from the left renal artery, and the end of a 5–0 nylon suture was placed above and below it. Next, the cold-stored donor newborn pig left kidney was positioned and the anastomosis was started with 5–0 nylon at the top and bottom margins of the Carrel patch site of the renal artery with continuous suture. The donor’s renal artery was 10 mm long and the diameter of Carrel patch was 10 mm. The transplanted kidney was lifted, and the posterior wall of the renal artery was sutured with continuous sutures. The anterior wall was then anastomosed. The anastomosis was completed within 15 min, the transplanted kidney artery was clamped with a vascular clamp, and the Satinsky clamp on the aorta was released to free the lower limb ischemia. The monkey’s left renal vein and the porcine graft’s left renal vein were then anastomosed with 6–0 or 7–0 nylon. The posterior wall was sutured with continuous suture, and the anterior wall was sutured with interrupted suture to restore blood flow to the transplanted kidney. The monkey’s left ureter and pig’s left ureter were anastomosed with 6 knotted 8–0 nylon sutures (Figs. 3a and b). These anastomoses were sutured using a x3 loupe. After carefully checking the outflow of the blood flow to the graft, the transplanted kidney was fixed with the peritoneum that covered the original kidney so that the position of the transplanted kidney would not shift. The surgery was completed after abdominal and wound closure.

The pig donor kidneys were cold-stored for 3 h, and the warm ischemia time was under approximately 40 min.

### Immunosuppressive, anti-inflammatory, and supportive therapy

Immunosuppressant therapy was administered as in a previous report^12^, with some modifications. Details of therapy are provided in Table 1.

**Table 1:** Immunosuppressive, anti-inflammatory, and adjunctive drugs used in this study.

Drugs	Dose	Days																	
		-9	-8	-7	-6	-5	-4	-3	-2	-1	ope	1	2	3	4	5	6	7	8
Thymoglobulin (ATG)	10 mg/kg (i.v. 3 h)							੦											
Anti-CD20mAb (Rituximab)	10 mg/kg (i.v. 3 h)								੦										
Abatacept	50 mg/kg (i.v. 0.5 h)										੦								
Tacrolimus	0.01 to 0.06 mg/kg x2/day (i.m.)	੦	੦	੦	੦	੦	੦	੦	੦	੦	੦	੦	੦	੦	੦	੦	੦	੦	੦
Mycophenolate mofetil	10 to 50 mg/kg x2/day (p.o.)					੦	੦	੦	੦	੦		੦	੦	੦	੦	੦	੦	੦	੦
Methylprednisolone	10 mg/kg (i.v.) 0.5 to 5 mg/kg/day (p.o.)									੦	੦	੦	੦	੦	੦	੦	੦	੦	੦
Tocilizumab	10 mg/kg (s.c.)								੦									੦	
Etanercept	0.5 mg/kg (s.c.)										੦				੦		੦		
Aspirin	40 mg/kg/every other day (p.o.)											੦		੦		੦		੦	
Low molecular weight heparin	700 U/body/day (s.c.											੦	੦	੦	੦	੦	੦		੦
Erythropoietin	2000 U/body/twice a week (s.c.)											੦			੦				੦
Famotidine	0.25 mg/kg x2/day (p.o.)											੦	੦	੦	੦	੦	੦	੦	੦
Valganciclovir	15 mg/kg/day (p.o.)					੦	੦	੦	੦	੦		੦	੦	੦	੦	੦	੦	੦	੦
Benzylpenicillin	95000 U/body/day (i.m.)										੦	੦	੦	੦	੦	੦	੦	੦	੦

### Blood flow assessment of the kidney transplants

The blood flow of the kidney transplants was assessed postoperatively under anesthesia by echography, and was reassessed by echography from the body surface with the recipient seated in a monkey chair, when immunosuppressant therapy was administered 8 days postoperatively. Blood flow was assessed by visual inspection and echography assessed with a SONIMAGE HS1 PRO^™^ (Konika Minolta) and probe C5-2 or HL18-4. Renal blood flow in the transplanted kidney was visually measured in Doppler echo mode by echography. One of the cases transplanted by the conventional method was not assessed by echography. The other case died in 7 days after surgery, so blood flow could not be assessed 8 days postoperatively, and it was made histopathologically.

## Results

**Figure 4:**
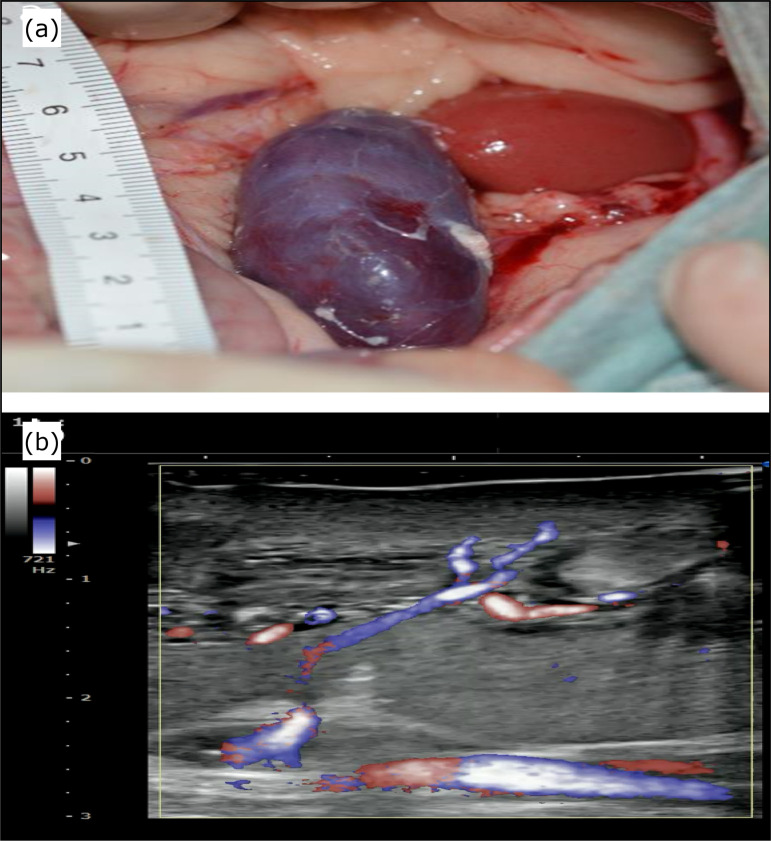
Pig kidney transplanted by the conventional method. **(a)** Photograph taken during the anastomosis. **(b)** Confirmation of blood flow in the transplanted kidney by Doppler echography.

**Figure 5:**
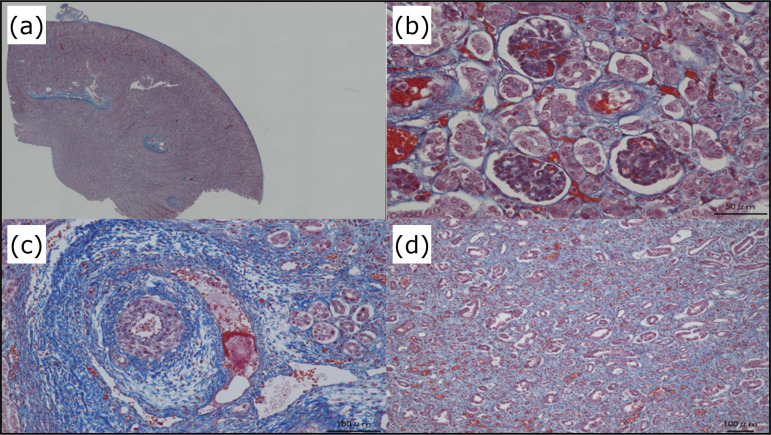
Pathology of the pig kidney transplanted by the conventional method. **(a)** Overall image. **(b)** Cortex. Slight collapse of the glomeruli are observed, but without clear internal thrombi. **(c)** Blood vessels. No findings of clear obstruction or endothelitis. **(d)** Medulla. Dissection from the basal membrane is observed in numerous tubules.

Among those with a kidney transplanted by the conventional method (two cases), venous perfusion after the restoration of the blood flow was macroscopically insufficient in all cases (Fig. 4a). In one case, the inflow artery and outflow vein were identified on echography after the vascular anastomosis (Fig. 4b). The postoperative recipient monkey often crouched down, and compression of the lower abdomen was observed. The general condition of the monkey deteriorated. The monkey died 7 days postoperatively and the transplanted kidney was removed shortly after death. Pathologically, there were no clear findings of thrombosis, vascular obstruction, or cell infiltration. Moreover, there were no findings of endoangiitis, which suggested that there was no hyperacute rejection observed in xenotransplantation without immunosuppression; rather, vascular insufficiency occurred, as indicated by the findings of glomerular collapse and dissection of the renal tubules from the basal membrane (Figs. 5a–d).

**Figure 6:**
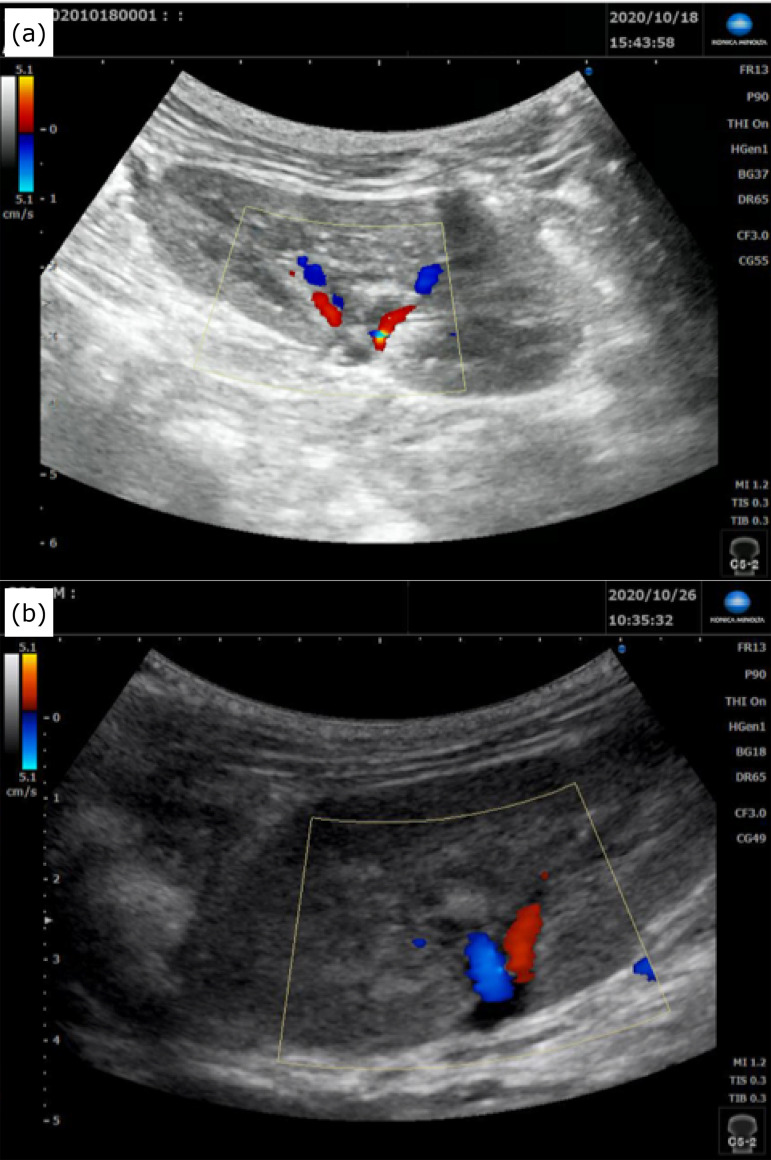
Blood flow of the transplanted kidney after orthotopic transplantation. **(a)** Immediately after transplantation. **(b)** One week later.

In contrast, the kidneys transplanted by our method showed no blood flow insufficiency in the early stage, as observed by echography in the acute postoperative stage (Figs. 6a and b).

## Discussion

We have previously conducted many pig-to-pig transplants by the conventional method, using the lower abdominal aorta^12^,^13^. After reperfusion to the graft in this study, we assessed the blood flow by echography and confirmed that there was blood flow in the renal arteries and veins. However, with the conventional method, the transplanted kidney turned dark red, and the presence of venous congestion was suspected. These findings and the postoperative course suggested that transplantsby the conventional method may be susceptible to torsion of the venous anastomosis due to body movements, which led us to decide on orthotopic transplantation to the natural locations of the renal artery and vein, which we previously reported in a pig-to-pig renal transplant^10^. The ingenuity of the orthotopic transplant method developed in this study lies in the use of the recipient’s renal artery and vein, rendered possible by performing the recipient’s kidney removal first. In order to do so, the diameter of the recipient renal artery anastomosis was adjusted so that the Carrel patch of the donor kidney could be attached accurately (Fig. 2). This resulted in the observation of visible blood flow on echography by Doppler echo mode (Figs. 6a and b).

This study comprised a xenotransplantation model of transplanting porcine kidneys to a non-human primate. Previous studies on similar xenotransplantations that have mentioned body sizes^4^-^7^,^14^ are summarized in Table 2. Among these studies, vascular complications were relatively rare in models that used large recipients, such as baboons, whereas more frequent when the recipients were smaller monkeys.

**Table 2:** Animal size and age in previous pig-to-primate renal transplants.

Donor	Recipient	Reference
18 to 40 kg (2 to 4 months)	Baboon 10 to 17 kg	Bühler *et al.* 5
18 to 40 kg (2 to 4 months)	Baboon 10 to 17 kg	Gollackner *et al*.^6^
16 to 18 kg (2 to 3 months)	Baboon 7 to 9 kg	Iwase *et al*.^7^
3.5 to 4.5 kg (3 to 4 weeks)	Monkey 3 to 5 kg (3 to 5 years)	Wang *et al*.^4^
6 to 14 kg (3 to 7 weeks)	Monkey 3 to 6 kg (3 to 4 years)	Spiezia *et al*.^14^

The current report focused on the transplantation techniques and assessed the presence of blood flow up to 8 days after surgery by echography. Long-term pathological findings of renal transplantation using the present improved operative model will be reported in a subsequent article, which will also describe transplants using genetically modified donor pigs. Moreover, the rejection of xenotransplants of organs with blood vessel attachment has been reported to occur after 2 weeks; thus, the development of immunosuppressant therapy that overcomes such rejections is anticipated.

## Conclusions

We achieved stable outcomes in a size-matched xenotransplant by orthotopic fixation of the donor kidney in the recipient. Although this report detailed only the surgical techniques in xenotransplants, we believe it provides valuable data for a monkey model with a smaller recipient size.
